# Mixing oak and eucalyptus sawdusts improves shiitake (*Lentinula edodes*) yield and nutritional value

**DOI:** 10.1371/journal.pone.0309787

**Published:** 2024-11-19

**Authors:** Zeina El Sebaaly, Stephanie Nabhan, Joelle Outayek, Teodor Nedelin, Youssef N. Sassine

**Affiliations:** 1 Department of Plant Production, Faculty of Agriculture, Lebanese University, Beirut, Lebanon; 2 Department of Agronomy, Faculty of Agronomy, University of Forestry, Sofia, Bulgaria; 3 Department of Forestry, Faculty of Forestry, University of Forestry, Sofia, Bulgaria; ICAR-Directorate of Mushroom Research, INDIA

## Abstract

The study aimed to explore suitable substrates comprising locally available hardwood sawdusts for the cultivation of Shiitake (*Lentinula edodes*) in Lebanon. Sawdusts of oak (OS), maple (MAP), and eucalyptus (EUC) were used alone or in combination, supplemented equally by wheat bran (WB). Results showed that complete mycelia run, fruiting, and harvest dates were the minimum in OS-WB: 800–200 by 72.2, 75.5, and 79.5 days after spawning (DAS) respectively, and the maximum in EUC-MAP-WB: 400-400-200 (by 88.3, 87.5, and 92.0 DAS, respectively). The substrate EUC-OS-WB: 400-400-200 had the highest biological efficiency (74.1%) compared to all treatments. Mushroom numbers ranged between 13.0 and 29.5 at harvest 1 (H1) and between 9.5 and 26.5 at harvest 2 (H2), showing a significant decrease in H2 in comparison to H1 in all treatments. Mushroom weight ranged between 8.8 and 25.9 at H1 and between 5.9 and 14.6 at H2. Furthermore, stepwise correlation showed that total biological yield (TBY) was positively affected by the biological yield at first harvest (BYH1) in OS-WB: 800–200 (R^2^ = 0.943), and at BYH2 in EUC-WB:800–200 (R^2^ = 0.944) and MAP-WB: 800–200 (R^2^ = 0.998), and it was negatively affected by BYH1 and stipe diameter in MAP-OS-WB: 400-400-200 (R^2^ = 0.946). Also, there was an improvement in mushroom protein, crude fibers, and vitamin C contents, and a decrease in carbohydrate contents on most substrates compared to control. Mushrooms obtained in EUC-OS-WB:400-400-200 recorded the highest protein and crude fiber contents (15.1 and 5.4%). Therefore, the mixture containing oak and eucalyptus sawdust has a good potential to improve shiitake yield and nutritional value compared to oak sawdust and could be an appropriate alternate for producing shiitake mushrooms.

## Introduction

*Lentinula edodes*, commonly known as shiitake mushroom originates from East Asia and is classified within the genus *Lentinula* and the order Agaricales [1]: “Shii” means the hardwood of *Castanopsis* spp., “take” means mushroom, and it is now the most popular name worldwide [2]. It is the second-most cultivated edible species in the world [3] and represents 25% of the world’s mushroom production [4]. Shiitake gained its popularity due to its medicinal and nutritional values, its unique flavor and distinguished by the ‘umami’ taste, which is becoming more in demand with the spread of Asian cuisine worldwide [5]. It grows naturally on dead logs of several hardwoods, primarily *Quercus* spp. (Oak) [6], and there are two ways to cultivate it: ‘natural logs’ and ‘synthetic logs’. The first method (natural logs) uses logs of evergreen hardwood, predominantly of the Fagaceae family [1]. However, because it requires a lot of land and trees, as well as a long cultivation cycle (around three to four years), it is considered expensive [7]. Therefore, contemporary growers have shifted towards cultivation using the ‘synthetic’ logs method. Comprising hardwood sawdust enriched with starch-based supplements (millet or wheat bran), these synthetic logs offer the potential for three to four times more mushroom yield in a shorter time period (around 4 months) compared to the natural logs [8, 9]. Because sawdust properties exert a significant impact on the growth and productivity of the cultivated mushrooms, it is imperative that they meet their nutritional requirements [10–12]. On synthetic logs, different types of sawdust (oak, maple, birch, *etc*.) are used in combination with starch-based supplements (wheat bran, rice bran, millet, rye, *etc*.) [13, 14], but oak (*Quercus* spp.) sawdust is the most frequently utilized at the commercial scale [15]. Normally, sawdusts are selected based on their availability in the production area [16]. For instance, shiitake growers may select cheap, easily available maple and eucalyptus woods [17], which have been found to be appropriate growing media for shiitake cultivation on ‘natural logs’ [18, 19]. On the other hand, there are still few studies concerning the cultivation of shiitake on ‘synthetic logs’ containing sawdust from maple or eucalyptus trees. Previous studies with eucalyptus sawdust and hardwood logs were conducted by Eira et al. [6] and Queiroz et al. [18], respectively, while studies with maple sawdust were done by Frey et al. [15] and Ranjbar and Olfati [20]. In Lebanon, the lack of know-how concerning shiitake cultivation on ‘synthetic logs’ has been hindering the expansion of this mushroom. Thus, the objective of this study was to determine the optimal substrate formula in terms of shiitake production, quality, and nutritional value, and to promote shiitake production in Lebanon. This was attempted by experimenting with shiitake (strain 3782) cultivation on ‘synthetic logs’ composed of locally available hardwoods such as oak (Quercus *libani*), eucalyptus (*Eucalyptus globulus*), and maple (*Acer monspessulanum ssp*. *microphyllum*) trees, either alone or in different combinations.

## Materials and methods

The study was conducted at the Agricultural and Veterinary Research Centre of the Lebanese University, Faculty of Agriculture Engineering and Veterinary Medicine, Ghazir Station.

### Experimental treatments

The experiment evaluated the effect of different substrate formulas (T1, T2, T3, T4, T5, and T6) containing oak sawdust (Quercus *libani*) (OS), maple sawdust (*Acer monspessulanum ssp*. *microphyllum*) (MAP), or eucalyptus sawdust (*Eucalyptus globulus*) (EUC). These species can be obtained through pruning carried out by municipalities and were selected because of their wide availability in Lebanon. Various species of wood logs were pruned at different times starting in the spring. Sawdusts of these species were used either alone or in combination and were supplemented equally with wheat bran: T1:OS-WB:800–200 (control), T2:EUC-WB:800–200, T3:MAP-WB:800–200, T4:EUC-OS-WB:400-400-200, T5:MAP-OS-WB:400-400-200, and T6:EUC-MAP-WB:400-400-200. The experimental design was a complete randomized design (CRD), with six treatments and six replicates per treatment (six bags of 1 kg capacity).

### Analytical tests on substrates

A sample of each sawdust type (OS, MAP, and EUC) was used for conducting the following analytical tests at the laboratories of the Lebanese Agricultural Research Institute (LARI)-Tal Amara station: Crude protein using the micro-Kjeldahl method (N × 6.25) [21]. Moisture content according to [22]: drying 2 g of substrate samples to a constant weight at 95–100°C under a pressure of 100 mm Hg. Ash content according to [22]: weighing 2 g of sample into a porcelain crucible, placing it in a temperature-controlled furnace preheated to 600°C for 2 hours, then leaving it to cool. The ash content (%) was calculated as follows:

$$Ash(\text{\%})=\frac{\text{Initial}\;\text{weight}\;\text{of}\;\text{sample}\;(\text{W}1)(\text{g})-\text{weight}\;\text{of}\;\text{sample}\;\text{after}\;\text{ash}\;\text{drying}\;(\text{W}2)(\text{g})}{\text{Initial}\;\text{weight}\;\text{of}\;\text{sample}\;(\text{W}1)\;(\text{g})}$$ (1)

Crude fat was determined by acid hydrolysis, according to AOAC [23]. Carbohydrates content was assessed by calculation:

Carbohydrates(%)=100−moisture(%)−crudeprotein(%)−crudefat(%)−minerals(%) (2)

Dry matter, organic matter, carbon, nitrogen, and carbohydrates contents of sawdust were determined by calculation as follows:

Drymatter(%)=100−moisture(%) (3)

Organicmatter(%)=100−ash(%) (4)

$$\text{Carbon}\;(\text{\%})=\frac{\text{organic}\;\text{matter}\;(\text{\%})}{1.72}$$ (5)

Nitrogen content was calculated from the following formula:

Crudeprotein(%)=Nitrogen(%)×6.25 (6)

Crude fibers were determined based on the Weende method [24]. Fiber fractions (acid detergent fiber: ADF, neutral detergent fiber: NDF, and acid detergent lignin: ADL) were measured by the Fibertherm methodology [25]. Then, the content of cellulose, hemicellulose, and lignin was calculated as follows:

Lignin(%)=ADF(%) (7)

Hemicellulose(%)=NDF(%)−ADF(%) (8)

Cellulose(%)=ADF(%)−ADL(%) (9)

Also, pH and electrical conductivity (EC) were measured in filtrates of substrate samples using a pH-meter (ADWA AD 132) and an EC-meter (ADWA AD 310).

### Substrate preparation and pasteurization

Wood logs of the different species were reduced to sawdust size (10–20 mm) using a modified Pro woodchipper. The milling time varied from one to two hours, depending on the initial size of the logs. Then 0.5 kg of each sawdust type was subjected to chemical analysis. Sawdusts were pasteurized using boiling water for a short period of time- 15 minutes- to get rid of dirt or insects that may be present. Afterward, they were spread on nylon sheets and sundried for three to five days depending on the substrate. Then, six kilograms of each substrate mixture were prepared by mixing the ingredients with wheat bran using a concrete mixer. Mixtures were then wetted to a 60–70% moisture level when the squeezed substrate expanded slowly and no dampness appeared on the hand [26]. Wetted substrates were filled in autoclavable polypropylene filter patch plastic bags (48x19.5x11.5 cm and 0.2-micron patch) of 1 kg each. Bags were subjected to steam sterilization for six hours at 120°C and 100 mbar in modified barrel steamers equipped with a closing pressuring lid, thermometer, pressure gauge, and faucet. The pasteurized bags were left to cool in a room set at 16°C to reduce the substrate temperature to below 25°C, which is the suitable temperature for mycelial growth [27].

### Spawning and incubation

Substrates were inoculated at their top with Shiitake 3782 strain spawn, procured from "Mushies" (an online store), at a 2% rate (20 g per 1 kg of substrate) in hygienic conditions. Spawned bags were labelled and incubated at 20–22°C with a relative humidity of 50–60%, and complete darkness. The incubation room’s temperature and relative humidity were monitored and ensured to be maintained constant using a thermometer (LUTRON HT-3007SD). During incubation, the progress of mycelial growth was monitored daily, and five stages were recorded in Days After Spawning (DAS): Stage 1 or mycelia run: substrate fully colonized by a thin layer of white hyphae; stage 2 or mycelia coat formation: the mycelia sheet hardens and covers the whole substrate surface; stage 3 or bump formation: clumps of mycelia develop into a popcorn shape; stage 4 or browning/pigmentation: the mycelia block develops a dark brown and dry outer protective layer, and stage 5 or coat hardening: the dark coat hardens [2].

### Fruiting and harvest

Fruit induction was done at the end of stage 5 of mycelial growth. To create a thermal shock, bags were opened and blocks soaked in ice for 24 hours [12]. Then, blocks were placed in the fruiting room at a temperature of 16°C, a relative humidity above 90%, and artificial light (2000 lux). Humidifiers (SANI-JET AIR 2836/P0) were used to increase the relative humidity in the fruiting room. Around two to three days after soaking, mushroom primordia or pinheads started to appear on blocks, marking the beginning of the fruit formation. The date of fruit formation in DAS. At this stage, blocks were watered three times a day until mushrooms grew to their final shape. Full development of mushrooms took place around four to five days after pinhead initiation depending on the substrate. Mushrooms were harvested when they had visible gills, opened caps, and slightly curled edges [28], and the harvest date was recorded as the number of days from spawning to harvest (in DAS). After the first harvest, blocks were incubated in darkness at a temperature of 22°C and a relative humidity of 60% for a further two weeks and were soaked again in ice for 24 hours to induce a second flush of mushrooms. Mushrooms were harvested twice (harvest 1: H1, harvest 2: H2). Average mushroom number was recorded for each harvest (MNH1, MNH2). Average mushroom weight (g) was calculated for each harvest (MWH1, MWH2) by dividing the total weight of mushrooms harvested by the number of bags per treatment. The biological yield (g/bag) corresponded to the total weight of mushrooms harvested per bag at harvest 1 (BYH1) and harvest 2 (BYH2). The total biological yield (TBY) corresponded to the summation of BYH1 and BYH2. The biological efficiency (BE) was calculated based on [29], as follows:

$$BE(\%)=\frac{total\;bio\;\log ical\;yield\;(g/bag)}{initial\;dry\;weight\;of\;substrate\;(g)\times 100}$$ (10)

### Analytical tests on mushrooms

The mushrooms physical characteristics were evaluated by measuring pileus diameter (PD), pileus length (PL), stipe diameter (SD), and stipe length (SL) using a sliding caliper on mushrooms obtained at the first harvest. Further analytical tests were conducted on representative fresh samples of each treatment. For instance, mushroom firmness was measured using a setamatic penetrometer (Stanhope-Seta) on five different points of the mushroom’s cap. Moisture content was evaluated using a moisture analyzer (M5-Thermo A64M). Ash content was determined by heating five grams of macerated mushroom in a muffle furnace (Carbolite Furnace OAF 10/1) at 550°C for 24 hours and calculating the ash content as follows:

Ash(g)=W2(g)−W1(g) (11)

Where W2: weight of the crucible containing ash, and W1: weight of an empty crucible

Fat content was determined using the Soxhlet apparatus technique [30]: 10 g of mushroom sample was macerated by sea sand and placed in the extractor. The empty bottom flask of the extractor was weighed (W1), filled with 500 mL of hexane, and then subjected to heat. The cycle was repeated several times for 12 hours, where hexane evaporated and extracted fat remained at the bottom of the flask. The flask containing extracted fat was weighed (W2). The fat content was determined as follows and expressed in percentage:

Fat(g)=W2(g)−W1(g) (12)

Crude fiber content (%) was determined after fat extraction based on AOAC [31] as the loss of ignition of dried residue remaining after digestion of the sample with 1.25% (w/v) H_2_HSO_4_ and 1.25% (w/v) NaOH. Crude protein content (N x 4.38) (%) was assessed using the macro-Kjeldhall method according to Reis et al. [32]. Carbohydrates content (%) was estimated by calculation:

Carbohydrates(%)=100−moisture(%)−crudeprotein(%)−crudefat(%)−minerals(%) (13)

The vitamin C content was determined as ascorbic acid (mg/100 g) titrametrically using 2.6 Dichloropheno-Indophenol methods [33]. A known weight of sample was grinded and mixed with 25 ml of a 5% metaphosphoric acid solution, and stirred for 30 min. The mixture was then filtered through Whatman No. 42 filter paper using a suction pump. Ten milliliters of the filtrate were pipetted into a 250 ml conical flask and titrated with 0.025% of 2.6 Dichlorophenol-Indophenol reagents. The amount of vitamin C in each extract was calculated using the following equation:

$$mg\;of\;ascorbic\;acid\;per\;100\;g=\frac{AxIxV1x100}{V2xW}$$ (14)

A = quantity of ascorbic acid (mg) reacting with 1 ml of 2.6 indophenol;

I = volume of indophenol (in ml) required for the completion for the titration with extract;

V_1_ = total volume of extract;

V_2_ = volume of extract used for each titration.

W = weight of the mushroom sample extracted

Vitamin D_2_ as ergocalciferol and vitamin D_3_ as cholecalciferol (in μg/100 g) were determined by high performance liquid chromatography (HPLC) according to Mattila et al. [34]. All tests concerning the mushroom’s nutritional components were performed in triplicate.

### Statistical analysis

Data analysis applied the one-way ANOVA, and means were compared by Duncan’s Multiple Range test at *P*_*value*_<0.05 using SPSS 25 program version 26. An independent sample t-test was applied to compare production between harvests 1 and 2. A Stepwise regression analysis evaluated the relationship between the total biological yield (dependent variable) and the productive indicators (MNH1, MNH2, MWH1, MWH2, BYH1, BYH2, PD, PT, SD, and SL) as independent variables.

## Results

Analysis of substrate properties (Table 1) showed that maple (MAP) and eucalyptus (EUC) sawdust had higher C/N ratios than oak sawdust (OS) (227.3, 214.9, and 109.8, respectively). On the other hand, OS was the richest in crude protein and ash (3.04 and 7.45%, respectively). Further, OS had the lowest amount of carbohydrates compared to EUC and MAP (80.01%, 86.16, and 86.29%, respectively). Lignin and cellulose contents were the highest in EUC and the lowest in MAP. Hemicellulose was remarkably higher in MAP than in OS and EUC (37.17, 9.13, and 3.57%, respectively). The pH was low in EUC (4.2), higher in OS (5.4), and the highest in MAP (6.6).

**Table 1:** Properties of initial substrates.

	OS	EUC	MAP
**Dry Matter (%)**	91.26	88.8	90.96
**Moisture (%)**	8.73	11.19	9.03
**Carbon (%)**	53.80	58.03	56.83
**Nitrogen (%)**	0.48	0.27	0.25
**C/N ratio**	109.8	214.9	227.3
**Organic matter (%)**	92.54	99.81	97.76
**Crude protein (%)**	3.04	1.73	1.59
**Ash content**	7.45	0.18	2.23
**Fat (%)**	0.74	0.71	0.84
**Carbohydrates (%)**	80.01	86.16	86.29
**NDF (%)**	77.18	82.60	87.84
**ADF (%)**	68.05	79.03	50.77
**ADL = Lignin (%)**	29.20	39.34	21.6
**Cellulose (%)**	38.84	39.68	29.15
**Hemicellulose (%)**	9.13	3.57	37.17
**EC (μS/cm)**	722	317	399
**pH**	5.4	4.2	6.6

OS: oak sawdust, EUC: eucalyptus sawdust, MAP: maple sawdust, NDF: neutral detergent fiber, ADF: acid detergent fiber, ADL: acid detergent lignin

### Mycelia run, fruiting, and harvest

Results in Table 2 showed that stage 1 was significantly delayed in T3 and T6 compared to T1 (by 3.3 and 2.6 days, respectively). The dates of consecutive stages (2, 3, 4, and 5), fruiting, and harvest were significantly delayed in T3, T5, and T6: by around 6.6, 13.0, and 16.1 days, respectively, for stage 5, by 7.8, 14.3, and 12.0 days for fruiting, and by 8.3, 14.7, and 12.5 days for harvest. On the other hand, the dates of consecutive mycelia growth stages, fruiting, and harvest in T2 and T4 were comparable to T1.

**Table 2:** Dates of mycelia growth stages, fruiting, and harvesting (in DAS) recorded at tested treatments.

Treatments	Stage 1	Stage 2	Stage 3	Stage 4	Stage 5	Fruiting date	Harvest date
**T1: OS-WB: 8–2**	15.7 ±1.0c	22.8 ±1.9c	37.3 ±2.7c	61.3 ±3.1b	72.2 ±2.1d	75.5 ±1.4d	79.5 ±1.5d
**T2: EUC-WB:8–2**	15.7 ±1.6c	24.3 ±1.2bc	39.3 ±3.8bc	63.8 ±5.8b	75.2 ±6.9cd	79.0 ±6.6cd	83.0 ±6.5cd
**T3: MAP-WB:8–2**	19.0 ±2.6a	27.8 ±3.2a	43.5 ±5.1ab	69.5 ±5.7a	78.8 ±6.9bc	83.3 ±6.3bc	87.8 ±6.0bc
**T4: EUC-OS-WB:4-4-2**	16.2 ±1.7bc	22.7 ±3.4c	36.7 ±2.7c	62.2 ±3.5b	72.7 ±3.8cd	76.2 ±3.0d	80.2 ±3.3d
**T5: MAP-OS-WB:4-4-2**	17.3 ±1.9abc	26.5 ±2.2ab	44.5 ±3.3a	72.8 ±4.8a	85.2 ±4.7a	89.8 ±4.5a	94.2 ±4.2a
**T6:EUC-MAP-WB:4-4-2**	18.3 ±1.2ab	26.2 ±2.5ab	43.3 ±4.2ab	72.3 ±4.2a	88.3 ±3.7ab	87.5 ±3.2ab	92.0 ±2.9ab

Means followed by different letters in the same column indicate a significant difference *at P value* <0.05. According to Duncan’s multiple range test. OS: oak sawdust, EUC: eucalyptus sawdust, MAP: maple sawdust, WB: wheat bran, stage 1: Mycelia run, stage 2: Mycelia coat formation, stage 3: Bump formation, stage 4: Browning/pigmentation, stage 5: Coat hardening.

### Shiitake production

**Fig 1:**
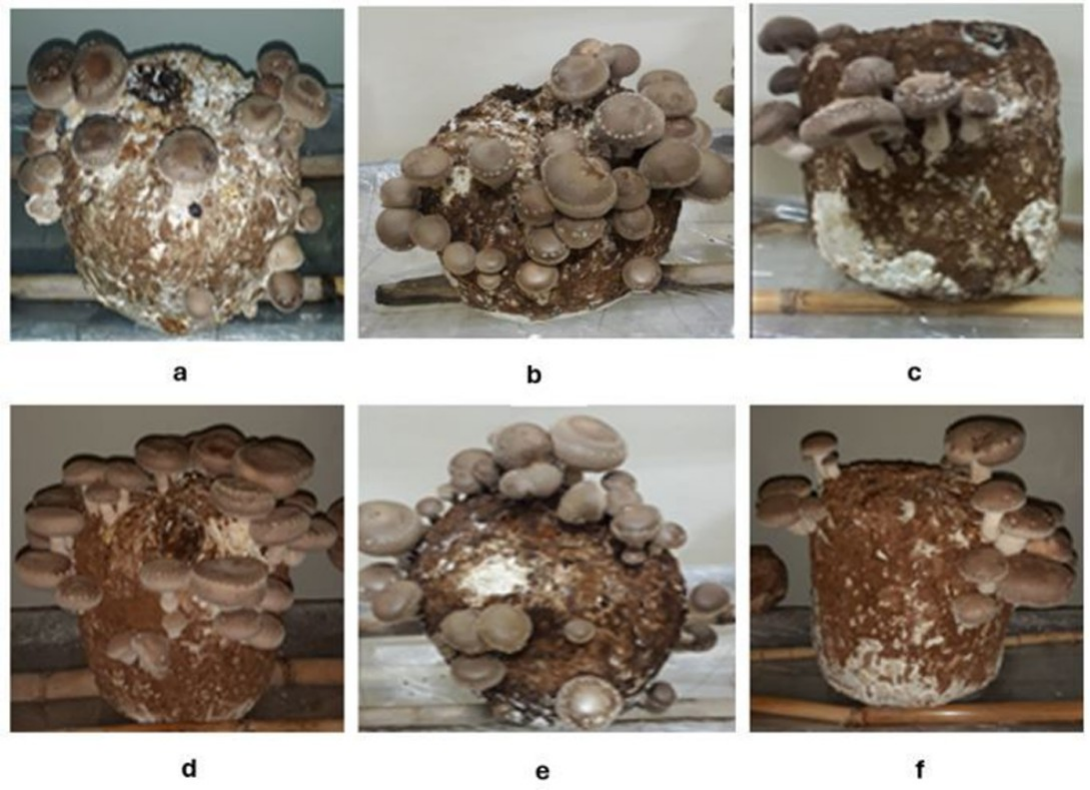
Fruiting blocks of the different treatments at the first flush. a: T1: OS-WB:800–200, b: T2: EUC-WB:800–200, c: T3: MAP-WB:800–200, d: T4: EUC-OS-WB:400-400-200, e: T5: MAP-OS-WB:400-400-200, f: T6: EUC-MAP-WB:400-400-200.

Results concerning productive indicators (Table 3) showed that at the first harvest, the average mushroom number was significantly higher in T4 and T5 (by 4.5 and 6.5 mushrooms, respectively) and significantly lower in T3 (by 16.5 mushrooms) than in T1. Mushroom weight was significantly higher than control in all treatments except T5. The average biological yield recorded at the first harvest (BYH1) was significantly higher than control in all treatments, recording a percentage improvement of around 35.8, 14.2, 29.5, 24.0, and 40.3% in T2, T3, T4, T5, and T6, respectively. Concerning the second harvest, the average mushroom number was significantly lower in T3 and T6 compared to all treatments. Compared to control, the average mushroom weight was significantly higher in T3 (by around 3 grams) and lower in T2, T5, and T6 (by around 2.8, 5.7, and 3.2 grams, respectively). The treatment T5 recorded a significantly lower mushroom weight compared to all treatments. The average biological yield obtained at harvest 2 showed a significant reduction in all treatments compared with control except in T4; the reduction was the most prominent in T6 (by around 64.6%). Nevertheless, the treatment T2 recorded a higher biological yield at harvest 2 compared with T3, T5, and T6. The total biological yield (TBY) in T4 was improved by around 19.5% compared to T1. On the other hand, it decreased significantly in T3 and was comparable with T1 in the treatments T2, T5, and T6. The highest biological efficiency was in T4 (74.1%), and the lowest in T3 (45.1%). The following Fig 1 shows the fruiting blocks at the first flush relative to each treatment.

**Table 3:** Productive indicators obtained at the first and second harvests (H1 and H2): Mushroom number (MN), mushroom weight (MW), biological yield (BY), and biological efficiency (BE).

Treatments	MN H1	MW H1 (g)	BY H1 (g/bag)	MN H2	MW H2 (g)	BY H2 (g/bag)	TBY (g/bag)	BE (%)
**T1: OS-WB:8–2**	29.5 ±3.5b	8.8 ±0.5e	275.0 ±52.2e	24.0 ±2.8a	11.6 ±3.3b	322.0 ± 43.7 a	597.0 ±93.2bc	59.7 ±9.3bc
**T2: EUC-WB:8–2**	29.3 ±3.6b	14.4 ±2.1c	428.6 ±18.8ab	24.8 ±2.8a	8.8 ±2.9c	221.4 ±74.5 b	649.9 ±62.5b	65.0 ±6.2b
**T3: MAP-WB:8–2**	13.0 ±2.1c	25.9 ±0.9a	320.4 ±3.1d	9.5 ±1.9c	14.6 ±0.5a	130.6 ±4.5 cd	451.0 ±7.6d	45.1 ±0.8d
**T4: EUC-OS-WB:4-4-2**	34.0 ±3.0a	11.7 ±1.5d	389.9 ±17.0bc	26.5 ±2.9a	13.9 ±2.2ab	351.5 ±22.9 a	741.4 ±36.8a	74.1 ±3.7a
**T5: MAP-OS-WB:4-4-2**	36.0 ±3.3a	9.8 ±0.9e	362.0 ±44.4c	26.5 ±3.4a	5.9 ±1.5d	162.6 ±6.3 c	524.6 ±89.9cd	52.5 ±9.0cd
**T6: EUC-MAP-WB:4-4-2**	28.3 ±3.0b	16.0 ±1.0b	461.1 ±38.3a	13.5 ±3.9b	8.4 ±0.6c	113.9 ±16.0d	575.0 ±108.9bc	57.5 ±10.9bc

Means followed by different letters in the same column indicate a significant difference at P value<0.05. according to Duncan’s Multiple range test. OS: oak sawdust, EUC: eucalyptus sawdust, MAP: maple sawdust, WB: wheat bran, mushroom number (MN), mushroom weight (MW), biological yield (BY), and biological efficiency (BE)

Results in Table 4 showed that there was a significant decrease in average mushroom number obtained in harvest 2 compared to harvest 1 at the level of all tested treatments. Further, mushroom weight decreased significantly in harvest 2 compared to harvest 1 in all treatments, except T1 and T4. Also, except in T1, biological yield decreased significantly at harvest 2; with the highest percentage decline in T6 (by 75%). Such a result reflects a consistency of production among consecutive harvests obtained on oak sawdust and a non-consistent production throughout the growing cycle in the remaining treatments.

**Table 4:** Productive indicators comparing harvests 1 and 2.

	T1:OS-WB:8–2	T2: EUC-WB:8–2	T3:MAP-WB:8–2	T4: EUC-OS-WB: 4-4-2	T5: MAP-OS-WB:4-4-2	T6: EUC-MAP-WB: 4-4-2
**MNH1**	29.5±3.5 a	29.3±3.6	13.0±2.1a	34.0±3.0a	36.0±3.3a	28.3±3.0a
**MNH2**	24.0±2.8b	24.8±2.8b	9.5±1.9b	26.5±2.88b	26.5±3.4b	13.5±3.9b
***P value***	0.014	0.035	0.012	0.001	0.001	0.000
**MWH1**	8.8±0.5 a	14.4±2.1a	26.0±0.9a	11.7±1.5a	9.8±0.9a	16.03±0.9a
**MWH2**	11.6±3.3a	8.8±2.9b	14.6±0.5b	13.9±2.2a	5.9±1.5b	8.4±0.6b
***P value***	0.066	0.003	0.000	0.073	0.000	0.000
**BYH1**	275.0 ±52.2a	428.6 ±18.8a	320.4 ±3.1a	389.9 ±17.0a	362.0 ±44.4a	461.1 ±38.3a
**BYH2**	322.0±43.7a	221.4±74.8b	130.6±4.5b	351.5±22.9b	164.2±5.1b	113.9±16.0b
***P value***	0.122	0.000	0.000	0.008	0.000	0.000

Means followed by different letters in the same column indicate a significant difference at P value<0.05. According to the independent sample t-test. MN H1: mushroom number in harvest 1, MN H2: mushroom number in harvest 2, MW H1: mushroom weight in harvest 1 (g), MW H2: mushroom weight in harvest 2 (g), BY H1: biological yield (g/kg) at harvest 1, BY H2: biological yield (g/kg) at harvest 2.

### Physical characteristics

The evaluation of the mushroom’s physical characteristics (Table 5) showed that compared to T1, the average pileus diameter (PD) was significantly higher in T6 (by 1.1 cm) and significantly lower in T2 and T5 (by 1.3 and 1.0 cm, respectively). The treatment T6 recorded significantly higher values of pileus diameter (PD), stipe diameter (SD), and stipe length (SL) compared to all other treatments. Furthermore, significantly lower SD was recorded in T2, T3, and T5 compared to control. Concerning the ratio PD/SL, it was lower than control in T2, T4, and T5. On the other hand, mushroom firmness did not differ significantly among all treatments.

**Table 5:** Mushrooms dimensions (pileus diameter: PD, pileus thickness: PT, stipe diameter: SD, stipe length: SL, ratio PD/SL, and firmness).

Treatments	PD (cm)	PT (cm)	SD (cm)	SL (cm)	PD/SL	Firmness (mm)
**T1: OS-WB: 8–2**	5.3±1.3b	1.2± 0.0a	0.9 ±0.2b	3.2±0.8bc	1.9±0.4a	5.2±0.8ab
**T2: EUC-WB: 8–2**	4.0±0.2d	1.1±0.1a	0.6±0.2c	2.7±0.2c	1.5±0.1b	5.3±1.0ab
**T3: MAP-WB: 8–2**	4.8±0.3bc	1.0±0.5a	0.6± 0.2c	2.8±0.6c	1.7±0.2ab	5.1±0.9ab
**T4: EUC-OS-WB:4-4-2**	4.9±0.1bc	1.2±0.05a	0.9±0.1b	3.3±0.2ab	1.5±0.2b	4.9±0.9b
**T5: MAP-OS-WB:4-4-2**	4.3±0.7cd	1.2±0.1a	0.6±0.1c	2.8±0.2c	1.6±0.3b	6.2±0.8a
**T6: EUC-MAP-WB:4-4-2**	6.4±0.5a	1.2±0.06a	1.1±0.2a	3.8±0.2a	1.7±0.1ab	5.5±0.9ab

Means followed by different letters in the same column indicate a significant difference at P value<0.05. According to Duncan’s multiple range test. OS: oak sawdust, EUC: eucalyptus sawdust, MAP: maple sawdust, WB: wheat bran.

Results of stepwise regression (Table 6) showed that there were positive correlations between total biological yield (TBY) and the biological yield obtained at harvest 1 (BY H1) in the treatment T1 (R^2^ = 0.943). In other terms, the higher the production obtained at the first harvest, the higher is the total production obtained on oak sawdust. Concerning the treatments T2, T3, and T4, the total biological yield was positively affected by the biological yield of harvest 2 (BY H2) (R^2^ = 0.944, R^2^ = 0.998, and R^2^ = 0.864, respectively). Thus, the higher the production maintained at the second harvest, the higher the total yield obtained from these substrates. On the other hand, in T5, there was a negative correlation between TBY, BYH1 and SD. Thus, a higher production at the first harvest will cause a decrease in the total production in the substrate composed of a mixture of maple and oak sawdust (MAP-OS-WB: 400-400-200). Mushroom mycelium utilizes the substrate’s available nutrients progressively during its growth [35]. Therefore, higher nutrient absorption from the first harvest may cause a subsequent reduction in available nutrients, negatively affecting the following harvest. Further, the largest stipe diameter in the same substrate was paired with the largest pileus diameter, indicating that bigger mushrooms were obtained at the first harvest. This led to a significant decrease in production at the second harvest and ultimately a reduced total biological yield.

**Table 6:** Significant correlations between the total biological yield (dependent variable) and productive indicators (independent variables) in the tested treatments (n = 6).

Treatments	Dep	Ind	Equation	ADJ R^2^
T1: OS-WB: 8–2	TBY	BY H1	117.39+ (0.977x BY H1)	0.943
T2: EUC-WB: 8–2	TBY	BY H2	468.314+ (0.977x BY H2)	0.944
T3: MAP-WB: 8–2	TBY	BY H2	232.003+ (0.999x BY H2)	0.998
T4: EUC-OS-WB: 4-4-2	TBY	BY H2	207.716+ (0.944x BY H2)	0.864
T5: MAP-OS-WB: 4-4-2	TBY	BY H1	-146.582+ (0.915x BY H1)	0.797
	TBY	BY H1, SD	-479.552+ (1.052x BY H1)+ (0.386x SD)	0.946

Dep: dependent variable, Ind: independent variables, TBY: total biological yield, BYH1: biological yield of harvest 1, BYH2: biological yield of harvest 2, SD: stipe diameter.

### Nutritional value

Results concerning the nutritional composition of mushrooms showed a significant increase in protein content in all treatments compared to T1. The treatment T4 recorded the highest value (15.1%). The crude fiber content showed a significant increase in T3, T4, and T6 compared to T1. The highest value of crude fiber was recorded in T4 (5.4%) and the lowest one in T1 (1.5%). A significant increase in fat content was recorded in T3 compared to T1 (0.4 and 0.3%, respectively). Also, the ash content showed a significant increase in T2, T3, and T6 compared to T1 (0.9, 1.0, 0.9, and 0.6%, respectively). Mushrooms harvested from all the treatments were poorer in carbohydrates compared to those of T1. Further, mushrooms of T5 were richer in carbohydrates compared with those of T2, T3, T4, and T6. Vitamin C content increased in mushrooms of T3, T4, T5, and T6 (8.0, 5.8, 8.5, and 7.3 mg/100g, respectively), while it decreased in mushrooms of T2 (4.0 mg/100g) compared to T1 (4.5 mg/100g). Finally, vitamin D2 and vitamin D3 showed similar values in all treatments (<3μg/L).

## Discussion

The date of the complete mycelia run on oak sawdust, which was recorded at 72 DAS, falls in the range that Chen [14] indicated for this stage on the same substrate, which is 30–120 DAS. On the other hand, this stage was delayed in all the treatments containing eucalyptus or maple sawdust, either alone or in combination. The non-fitting substrate pH (4.2 and 6.6, respectively) to the ideal range of 4.5 to 5.5 for mycelia formation [1, 36] and the lower ash content of maple and eucalyptus sawdust compared to oak sawdust might be responsible for this delay. According to Chen [14], the abundance of minerals in the growing substrate positively influences mycelia run and fruit formation. Furthermore, when compared to eucalyptus, maple, and their mixture, oak sawdust has the lowest C/N ratio, which could explain the faster development of shiitake on the latter substrate. According to Bellettini et al. [37] and Desisa et al. [38], lower C/N ratios are favored for a faster mycelia development of shiitake due to the higher bioavailability of nitrogen and its easy and fast assimilation by the fungus. The complete mycelia run and fruiting stages were the slowest on maple sawdust, supporting the findings of Ranjbar and Olfati [20]. The reason for this delay could be that maple sawdust needs more time to decompose because it contains more hemicellulose and less cellulose.

Kurt and Büyükalaca [39] and Xiao et al. [40] stated that mycelium secretes ligninases first, which break down lignin, followed by hemicellulases and cellulases, which break down holocellulose (hemicellulose and cellulose), which is the main source of carbon and energy required for mushroom growth. Also, there is a negative correlation between the amount of hemicellulose in the growing media and the growth of mycelia, as demonstrated by Ratnanindha et al. [41]. Moreover, compared to the first flush, the number of mushrooms on maple sawdust decreased, and the average weight of mushrooms on this sawdust type was higher than that of oak sawdust. Therefore, the decline in TBY may be attributed to the decrease in the number of mushrooms on maple sawdust. These results confirmed earlier findings of Ranjbar and Olfati [20Ranjbarandolfati2016], who reported a lower number of mushrooms, yield, and biological efficiency on maple sawdust compared to oak sawdust. Also, Bruhn et al. [42] obtained higher mushroom weight on sugar maple logs than on oak logs. Early studies on shiitake indicated biological efficiencies of 79.5% on maple sawdust [20Ranjbarandolfati2016] and 80.7% on oak sawdust [43].

Further, spawn run was delayed when eucalyptus sawdust was used alone, while oak and eucalyptus sawdust mixed together showed simultaneous timing with oak sawdust. In comparison to oak sawdust, the biological efficiency was slightly higher on eucalyptus sawdust and significantly higher on the mixture of oak and eucalyptus sawdust. These results corroborated those of Kannan [44], who noted a higher production and a more rapid spawn run on silver oak sawdust compared to eucalyptus sawdust or a combination of the two sawdust types. In comparison to oak sawdust, the substrates combining oak and eucalyptus sawdust (T4) or oak and maple (T5) showed a slight or significant increase in the number and weight of mushrooms. This suggests a tendency to improve these indicators of productivity at subsequent harvests.

Therefore, an increase in the C/N ratio of the substrate might not always have a negative effect on total production. Ultimately, biological efficiencies of mixtures combining oak sawdust with maple or eucalyptus (52.5% and 74.1%) at high C/N ratios were either significantly higher or comparable to that obtained from oak sawdust (59.7%) at low C/N ratios. At an industrial scale, farmers can harvest 0.3 to 0.5 kg of shiitake mushrooms from one kilogram of dried substrate (BE: 30–50%) [14].

Early research on shiitake mushrooms indicated dimensions that were similar to those found in this study. The ranges were 5.1–6.0 cm for pileus diameter, 1.0–1.3 cm for pileus thickness, 2.7–4.5 cm for stipe length [45, 46]. A greater mushroom firmness in the mushrooms obtained on a mixture of oak and maple sawdust (T5), is an indication of higher-quality mushrooms. Firmer pileus is preferable among consumers because it gives the mushroom a more substantial bite, especially when it is intended to be eaten as a whole [47].

The strain of mushrooms being produced as well as the substrates utilized to cultivate them have a significant effect on their nutritional composition [48–52]. The values obtained for the protein content of shiitake cultivated on the different substrates ranged from 2.7 to 15.1%. These values were below both the range of 12.4–17.2% reported by Gaitán-Hernández et al. [53] and the range of 15.4–22.0% found by Morais et al. [54] for mushrooms cultivated on rice straw. Queiroz et al. [18] reported a protein range of 20.1–22.6% on eucalyptus logs, while Baktemur et al. [46] obtained a protein content of 21.2% on oak sawdust. These values were higher than those obtained in the present study. All of the investigated substrate types did, however, result in a higher protein content than that which was obtained on oak sawdust, the substrate that is commonly used. A healthy diet must contain proteins because they are the major structural component of muscle and other tissues in the body and because they are necessary for the synthesis of hormones, enzymes, and hemoglobin [55].

Proteins are crucial constituents of the dry matter of the mushroom, they can be used to bridge the protein malnutrition gap [56]. The range of fiber content found in mushrooms was 1.5–5.4%, which was less than the range indicated by Queiroz et al. [18] (11.3–14.2%) and Desisa et al. [38] (13.9–18.0%). Further, mixing oak and eucalyptus sawdust (T4) caused a significant improvement in the fiber content of mushrooms. According to Sheng et al. [57], mushroom fiber is mostly soluble fiber and can be absorbed by humans. Fiber helps with reducing obesity, managing diabetes, improving gut health, lowering cholesterol, enhancing immunity, and treating constipation. From the view of modern nutrition, fiber is becoming a vital component of food [58]. Moreover, shiitake mushrooms grown on all of the tested substrates have vitamin C contents ranging from 4.0 to 8.5 mg/100 g, which was a higher range than that reported by Breene. [59]: 2.1–1.6 mg/100 g. Fresh shiitake mushrooms provided 8.24 mg/100 g of vitamin C, according to Stojanova and Karakashov [60]. In addition to its antioxidant and anti-inflammatory properties, vitamin C is essential for maintaining adequate bone density, helps in stress control, and plays an important role in immune system maintenance [61].

## Conclusion

The study provided appropriate lignocellulosic substrates, which are abundant in Lebanese forests, for the cultivation of shiitake mushrooms. A substrate consisting of equal amounts of eucalyptus and oak sawdust can substitute oak sawdust, causing a close spawn run duration, consistent production across subsequent harvests, superior biological yield, and better nutritional value (higher protein, crude fibers, and vitamin C). On the other hand, in order to attain good results, employing eucalyptus and maple sawdust alone will require a higher supplement dose. In conclusion, the research offered valuable guidance for Lebanese mushroom growers regarding the optimal substrate choices for future shiitake investments.

## Supporting information

S1 Raw data (PDF)
