# Supplementary material for: Mixing oak and eucalyptus sawdusts improves shiitake (Lentinula edodes) yield and nutritional value
Source: PLoS One. 2024 Nov 19;19(11):e0309787. doi: 10.1371/journal.pone.0309787 (PMC11575786; doi:10.1371/journal.pone.0309787)
Supplement: S1 Raw data — (PDF) [file pone.0309787.s001.pdf]

Raw data used for testing the mycelial growth

Treatment	Stage 1	stage 2	Stage 3	Stage 4	Stage 5	fruiting	harvest
OS-WB: 800-200	16.0	21.0	35.0	58.0	70.0	75.0	79.0
OS-WB: 800-200	15.0	25.0	41.0	64.0	73.0	76.0	80.0
OS-WB: 800-200	16.0	24.0	35.0	60.0	74.0	77.0	82.0
OS-WB: 800-200	17.0	20.0	40.0	63.0	71.0	74.0	78.0
OS-WB: 800-200	14.0	24.0	38.0	65.0	70.0	74.0	78.0
OS-WB: 800-200	16.0	23.0	35.0	58.0	75.0	77.0	80.0
EUC-MAP-WB: 400-400-200	17.0	28.0	48.0	78.0	89.0	92.0	96.0
EUC-MAP-WB: 400-400-200	18.0	23.0	40.0	68.0	81.0	86.0	91.0
EUC-MAP-WB: 400-400-200	19.0	24.0	38.0	67.0	78.0	83.0	88.0
EUC-MAP-WB: 400-400-200	17.0	29.0	46.0	74.0	83.0	86.0	90.0
EUC-MAP-WB: 400-400-200	20.0	28.0	47.0	75.0	84.0	88.0	93.0
EUC-MAP-WB: 400-400-200	19.0	25.0	41.0	72.0	85.0	90.0	94.0
EUC-WB: 800-200)	17.0	25.0	43.0	68.0	80.0	83.0	87.0
EUC-WB: 800-200)	16.0	25.0	39.0	65.0	78.0	81.0	86.0
EUC-WB: 800-200)	18.0	26.0	45.0	72.0	84.0	88.0	91.0
EUC-WB: 800-200)	15.0	23.0	37.0	63.0	74.0	78.0	83.0
EUC-WB: 800-200)	14.0	23.0	36.0	58.0	70.0	75.0	78.0
EUC-WB: 800-200)	14.0	24.0	36.0	57.0	65.0	69.0	73.0
MAP-OS-WB: 400-400-200	20.0	27.0	45.0	77.0	90.0	94.0	99.0
MAP-OS-WB: 400-400-200	17.0	27.0	46.0	74.0	85.0	89.0	93.0
MAP-OS-WB: 400-400-200	16.0	29.0	47.0	78.0	89.0	94.0	98.0
MAP-OS-WB: 400-400-200	17.0	25.0	40.0	68.0	80.0	85.0	90.0
MAP-OS-WB: 400-400-200	15.0	23.0	41.0	66.0	79.0	84.0	89.0
MAP-OS-WB: 400-400-200	19.0	28.0	48.0	74.0	88.0	93.0	96.0
EUC-OS-WB : 400-400-200	14.0	19.0	33.0	59.0	69.0	72.0	76.0
EUC-OS-WB : 400-400-200	15.0	20.0	35.0	60.0	70.0	75.0	79.0
EUC-OS-WB : 400-400-200	18.0	25.0	39.0	65.0	78.0	80.0	84.0
EUC-OS-WB : 400-400-200	15.0	20.0	35.0	61.0	69.0	74.0	77.0
EUC-OS-WB : 400-400-200	17.0	27.0	40.0	68.0	75.0	78.0	83.0
EUC-OS-WB : 400-400-200	18.0	25.0	38.0	60.0	75.0	78.0	82.0
MAP-WB: 800-200)	21.0	32.0	50.0	75.0	88.0	92.0	97.0
MAP-WB: 800-200)	16.0	23.0	38.0	65.0	74.0	79.0	84.0
MAP-WB: 800-200)	23.0	30.0	48.0	76.0	86.0	89.0	93.0
MAP-WB: 800-200)	19.0	27.0	38.0	62.0	72.0	77.0	82.0

MAP-WB: 800-200)	18.0	29.0	42.0	67.0	73.0	78.0	83.0
MAP-WB: 800-200)	17.0	26.0	45.0	72.0	80.0	85.0	88.0

Raw data used for testing the productivity

Treatment	FN F1	FW F1	BY F1	FN F2	FW F2	BYF2	TBY	BE
OS-WB: 800-200	36.0	8.2	202.9	29.0	8.1	290.3	493.2	49.3
OS-WB: 800-200	31.0	8.4	345.2	21.0	16.7	390.7	735.9	73.6
OS-WB: 800-200	28.0	8.8	320.5	22.0	14.5	343.5	664.0	66.4
OS-WB: 800-200	27.0	9.1	240.5	25.0	9.3	280.5	521.0	52.1
OS-WB: 800-200	27.0	9.7	280.6	23.0	10.8	340.6	621.2	62.1
OS-WB: 800-200	28.0	8.6	260.5	24.0	10.3	286.3	546.8	54.7
EUC-MAP-WB: 400-400-200	33.0	14.5	413.4	13.0	8.3	103.3	605.8	60.6
EUC-MAP-WB: 400-400-200	26.0	16.7	490.5	15.0	8.1	105.9	606.1	60.6
EUC-MAP-WB: 400-400-200	30.0	15.5	418.7	9.0	8.8	99.5	494.5	49.4
EUC-MAP-WB: 400-400-200	25.0	17.1	502.5	17.0	8.0	113.5	413.7	41.4
EUC-MAP-WB: 400-400-200	27.0	16.5	486.3	18.0	7.8	143.6	598.9	59.9
EUC-MAP-WB: 400-400-200	29.0	15.9	455.3	9.0	9.5	117.8	731.0	73.1
EUC-WB: 800-200)	25.0	17.2	445.0	28.0	4.9	108.9	553.9	55.4
EUC-WB: 800-200)	31.0	13.5	410.5	21.0	12.1	244.6	655.1	65.5
EUC-WB: 800-200)	28.0	14.0	435.7	22.0	11.3	287.6	723.3	72.3
EUC-WB: 800-200)	34.0	11.8	400.3	25.0	10.4	310.2	710.5	71.1
EUC-WB: 800-200)	32.0	13.2	445.5	27.0	6.1	195.3	640.8	64.1
EUC-WB: 800-200)	26.0	16.5	434.4	26.0	8.0	181.5	615.9	61.6
MAP-OS-WB: 400-400-200	41.0	8.6	396.7	23.0	7.8	157.0	652.7	65.3
MAP-OS-WB: 400-400-200	37.0	9.5	360.4	25.0	6.7	166.2	529.6	53.0
MAP-OS-WB: 400-400-200	33.0	10.2	325.7	32.0	4.1	160.8	460.0	46.0

MAP-OS-WB: 400-400-200	35.0	9.9	414.2	26.0	5.5	172.0	585.2	58.5
MAP-OS-WB: 400-400-200	32.0	11.1	296.3	29.0	4.3	164.7	397.7	39.8
MAP-OS-WB: 400-400-200	38.0	9.3	378.6	24.0	7.0	164.7	522.5	52.2
EUC-OS-WB : 400-400-200	32.0	12.5	375.2	22.0	17.1	355.8	731.0	73.1
EUC-OS-WB : 400-400-200	37.0	10.5	369.2	27.0	13.3	308.0	677.2	67.7
EUC-OS-WB : 400-400-200	30.0	13.8	400.3	26.0	14.6	348.4	748.7	74.9
EUC-OS-WB : 400-400-200	38.0	9.5	410.0	29.0	12.0	367.9	777.9	77.8
EUC-OS-WB : 400-400-200	34.0	11.7	404.0	30.0	11.2	371.3	775.3	77.5
EUC-OS-WB : 400-400-200	33.0	12.4	380.6	25.0	15.4	357.6	738.2	73.8
MAP-WB: 800-200)	16.0	25.8	323.9	9.0	15.1	135.6	459.5	46.0
MAP-WB: 800-200)	11.0	26.5	316.8	11.0	14.0	125.9	442.7	44.3
MAP-WB: 800-200)	12.0	27.3	317.9	12.0	14.2	126.4	444.3	44.4
MAP-WB: 800-200)	15.0	25.6	320.0	7.0	15.1	130.2	450.2	45.0
MAP-WB: 800-200)	11.0	26.0	319.5	8.0	14.8	128.9	448.4	44.8
MAP-WB: 800-200)	13.0	24.6	324.2	10.0	14.5	136.4	460.6	46.1

Raw data used to test the mushroom composition

Treatment	PD F1	PL F1	SD F1	SL	PD/SL	firmness
OS-WB: 800-200	3.8	1.2	0.7	2.2	1.9	5.4
OS-WB: 800-200	6.7	1.2	1.1	3.6	2.3	5.38
OS-WB: 800-200	6.7	1.2	1.1	3.6	2.3	4.3
OS-WB: 800-200	3.8	1.2	0.7	2.2	1.9	4.15
OS-WB: 800-200	5.4	1.2	0.9	3.7	1.5	5.5
OS-WB: 800-200	5.4	1.2	0.9	3.7	1.5	6.2
EUC-MAP-WB: 400-400-200	6.5	1.1	1.0	3.8	1.7	6.1
EUC-MAP-WB: 400-400-200	6.8	1.2	1.0	3.9	1.7	5.6
EUC-MAP-WB: 400-400-200	5.8	1.3	1.2	3.8	1.5	5.6
EUC-MAP-WB: 400-400-200	7.0	1.2	1.3	4.0	1.8	6.5
EUC-MAP-WB: 400-400-200	6.7	1.2	0.9	3.6	1.9	4.1
EUC-MAP-WB: 400-400-200	5.8	1.2	1.1	3.5	1.7	4.9
EUC-WB: 800-200)	3.8	1.2	0.7	2.7	1.4	6.6
EUC-WB: 800-200)	4.3	1.0	0.5	2.9	1.5	5.5
EUC-WB: 800-200)	4.1	0.9	0.6	3.0	1.4	4.0
EUC-WB: 800-200)	3.7	1.1	0.6	2.5	1.5	4.7
EUC-WB: 800-200)	3.9	1.3	0.8	2.5	1.6	6.4
EUC-WB: 800-200)	4.2	1.1	0.4	2.7	1.6	4.8
MAP-OS-WB: 400-400-200	3.9	1.2	0.7	2.7	1.4	6.9
MAP-OS-WB: 400-400-200	3.6	1.3	0.6	2.8	1.3	5.1
MAP-OS-WB: 400-400-200	4.6	1.3	0.7	3.1	1.5	7.2
MAP-OS-WB: 400-400-200	4.2	1.0	0.5	2.9	1.4	5.6
MAP-OS-WB: 400-400-200	5.6	1.1	0.6	2.6	2.2	6.5

MAP-OS-WB: 400-400-200	4.1	1.4	0.5	2.5	1.6	6.0
EUC-OS-WB : 400-400-200	4.9	1.3	0.8	3.1	1.7	4.7
EUC-OS-WB : 400-400-200	5.0	1.2	0.9	3.6	1.4	6.4
EUC-OS-WB : 400-400-200	4.7	1.2	0.9	3.3	1.4	4.8
EUC-OS-WB : 400-400-200	4.9	1.3	0.8	3.1	1.7	4.1
EUC-OS-WB : 400-400-200	4.7	1.2	0.9	3.3	1.4	4.0
EUC-OS-WB : 400-400-200	5.0	1.2	0.9	3.6	1.4	5.4
MAP-WB: 800-200)	5.0	1.1	0.5	2.5	2.0	5.9
MAP-WB: 800-200)	4.0	0.6	0.4	2.3	1.7	6.2
MAP-WB: 800-200)	4.0	0.6	0.5	3.0	1.3	4.3
MAP-WB: 800-200)	5.2	1.9	1.0	3.8	1.4	5.6
MAP-WB: 800-200)	4.6	1.1	0.5	2.6	1.8	4.3
MAP-WB: 800-200)	3.8	0.9	0.5	2.3	1.7	4.2
